# Unsupervised learning reveals rapid gait adaptation after leg loss and regrowth in spiders

**DOI:** 10.1242/jeb.250243

**Published:** 2025-06-17

**Authors:** Suzanne Amador Kane, Brooke L. Quinn, Xuanyi Kris Wu, Sarah Y. Xi, Michael F. Ochs, S. Tonia Hsieh

**Affiliations:** ^1^ Department of Physics and Astronomy, Haverford College, Haverford, PA 19041, USA; ^2^ Department of Biology, Temple University, Philadelphia, PA 19122, USA; ^3^ Department of Mathematics and Statistics, The College of New Jersey, Ewing, NJ 08628, USA

**Keywords:** Autotomy, Arachnid, Clustering, Locomotion, Robustness, Stability

## Abstract

Many invertebrates voluntarily lose (autotomize) limbs during antagonistic encounters, and some regenerate functional replacements. Because limb loss can have severe consequences on individual fitness, it is likely subject to significant selective pressures, making this an excellent phenomenon with which to investigate biomechanical robustness. Spiders frequently autotomize one or more legs. We investigated the time course of locomotor recovery after leg loss and regeneration in juvenile tarantulas (Arachnida: Araneae) naive to autotomy. We recorded high-speed video of spiders running with all legs intact, then immediately after, and again 1 day after they had autotomized two legs. The legs were allowed to regenerate, and the same sequence of experiments was repeated. Video tracking analysis revealed that the spiders resumed their pre-autotomy speed and stride frequency after leg regeneration and in ≤1 day after both autotomies; path tortuosity was unaffected by these treatments. Autotomized spiders widened the spread of their remaining legs for stability and to compensate for missing functional space. To analyze how their gaits changed in response to leg loss, we applied unsupervised machine learning for the first time to measured kinematic data in combination with gait space metrics. Spiders were found to robustly adopt new gait patterns immediately after losing legs, with no evidence of learning. This novel clustering approach both demonstrated concordance with hypothesized gaits and revealed transitions between and variations within these patterns. More generally, clustering in gait space enables the identification of patterns of leg motions in large datasets that correspond to either known gaits or undiscovered behaviors.

## Teaser

**Highlighted Article:** Unsupervised learning and kinematic analyses revealed that spiders with two amputated legs recovered normal running performance after leg regeneration and ≤1 day of limb loss, adopting new gaits and postures to recovery robustly without learning.

## INTRODUCTION

Animals have a remarkable ability to navigate effectively through complex and unpredictable natural environments, sometimes even after suffering significant injuries such as limb loss. In particular, many invertebrates will voluntarily amputate (i.e. autotomize) limbs in response to limb injury or entrapment, predator attack, intraspecific antagonistic encounters and failed molting (Emberts et al., 2019; Fleming et al., 2007). Because limb loss may have severe consequences for individual fitness, there are likely strong selective pressures for rapid recovery from and robust locomotor adaptation to this perturbation. Indeed, significant performance decrements (e.g. reduced speed, moving in more tortuous trajectories) have been measured for many organisms missing limbs (Fleming et al., 2007; Han et al., 2024; Lutzy and Morse, 2008; Meshkani et al., 2023; O'Neil et al., 2024; Pfeiffenberger and Hsieh, 2021; Prestholdt et al., 2022; Escalante and O'Brien, 2024). Therefore, limb autotomy is a fruitful phenomenon with which to investigate biomechanical robustness in a tractable system in which this behavior is often a natural and commonplace occurrence (Emberts et al., 2019; Fleming et al., 2007; Mongeau et al., 2024).

How an animal's running performance depends on time after autotomy provides a crucial test of how it compensates for limb loss (Jayaram, 2015; Saintsing, 2022). If the organism uses adaptive control (i.e. error-based learning), then we expect its locomotor performance metrics (e.g. speed, acceleration, stability) to recover gradually as a function of time. Conversely, for robust control, we expect a rapid recovery as a result of near-immediate adoption of a new stable gait (Mongeau et al., 2024). To date, studies of recovery from limb autotomy in arthropods have been consistent with robust control; for example, the aquatic insect *Microvelia*, *Prionostemma* harvestmen and the cockroach *Blatta orientalis* were found to resume their pre-autotomy speed and other locomotor measures 1, 2 and 3 days, respectively, after leg loss (Escalante et al., 2020; Hughes, 1957; O'Neil et al., 2024). However, to our knowledge, post-autotomy locomotor recovery has not been measured for naive individuals that never previously experienced leg loss, nor has it been compared for successive autotomy treatments. These study designs could illuminate whether learning from experience promotes faster recovery.

Some studies have also considered the specific gait adaptions animals use to accommodate missing legs. Although some arthropods (*Microvelia* and cockroaches) resume their pre-autotomy gaits after an initial period of variable leg motions (Hughes, 1957; O'Neil et al., 2024), others (e.g. stinkbugs, harvestmen, spiders, scorpions and crabs) restore stable locomotion by adopting new gaits (Bowerman, 1975; Escalante et al., 2020; Han et al., 2024; Herreid and Full, 1986; Pfeiffenberger and Hsieh, 2021; Wilshin et al., 2018; Wilson, 1967). Some insects also modify their body and leg postures and motions to fill in missing functional space owing to lost legs (Han et al., 2024; Jayaram, 2015; Meshkani et al., 2023; Saintsing, 2022; Zeng et al., 2024). However, to our knowledge, there have been no studies that map quantitatively the detailed time course of recovery of gait leg motions and posture after such treatments. Furthermore, although the behavioral impact of limb regeneration after autotomy has been studied with respect to foraging, predation, spider web construction, sensing and mating (Maginnis, 2006), only one study of crabs has explored its impact on locomotion (Prestholdt et al., 2022).

In the present study, we addressed these knowledge gaps by measuring how preferred gaits changed over time following leg autotomy in spiders (Arachnida, order Araneae). Leg loss occurs frequently in this order of arachnids, with 5–40% of specimens observed in the field missing one or more limbs (Brown et al., 2018; Brueseke et al., 2001; Johnson and Jakob, 1999; Wrinn and Uetz, 2007). Limb loss might be expected to impede a spider's ability to capture prey and evade predators while navigating diverse, complex habitats. In practice, the loss of one or more legs was found to result in significantly reduced running speed for spiders in some (Apontes and Brown, 2005; Brown and Formanowicz, 2012; Gerald et al., 2017), but not all (Boehm et al., 2021; Brueseke et al., 2001; Wilshin et al., 2018), previous studies. Moreover, juveniles of many species can regenerate functional legs after molting (Foelix, 2011), enabling those spiders capable of surmounting the costs of autotomy to regain full locomotor function at a later time.

We investigated (1) whether and how spiders compensate for the loss of one or more limbs by changing their preferred gaits during locomotor recovery, (2) how leg regeneration influences locomotor performance in spiders and (3) whether spiders respond differently to subsequent repeated limb loss after regeneration. Our study organism was the Guatemalan tiger rump tarantula (*Davus pentaloris*, family Theraphosidae), selected for its rapid running – allowing us to assess the time course of recovery from injury and learning during execution of a high-performance behavior – and large size, permitting detailed tracking of their pose on video*.* In addition, tarantulas are known to autotomize legs restrained or damaged by predators, females during mating and incomplete molting (Foelix, 2011; Montes de Oca and Mendoza, 2020; Pizzi, 2006; Roth and Roth, 1984).

**Fig. 1.**
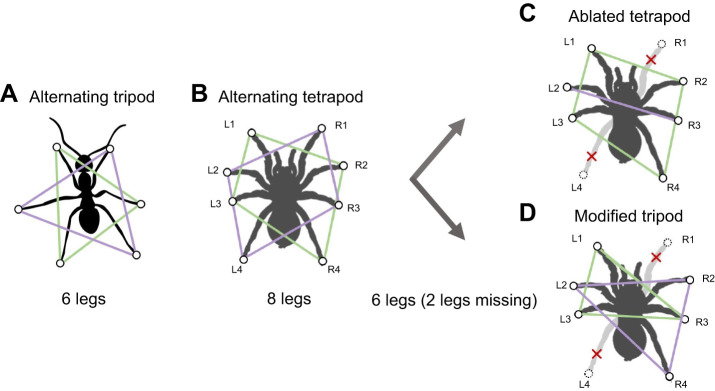
**Illustration of typical gaits used by hexapods (e.g. six-legged insects) and octopods (e.g. eight-legged spiders).** (A,B) The two sets of legs moved synchronously – (A) tripods of three legs for hexapods and (B) tetrapods of four legs for octopods – are indicated by green and purple lines, respectively. Gaits used by spiders that have lost two legs include (C) the ablated tetrapod, in which one tetrapod continues the same motion as pre-autotomy (green lines) and a bipod consisting of the other two intact legs move together (purple line), and (D) the modified tripod, in which the remaining intact six legs move in two alternating tripods, each of which incorporates legs from both original tetrapods. The labels used to denote spider legs in this study are indicated in B–D. The ant silhouette in A is from https://commons.wikimedia.org/wiki/File:Fourmi02.svg#file by Meul, accessed 2 May 2024 with a Creative Commons Attribution-Share Alike 3.0 Unported license.

Just as six-legged insects often locomote using the alternating tripod gait (Full and Tu, 1991), tarantulas and other spiders run using a gait in which two tetrapods (sets of four diagonally opposed alternating legs) move in synchrony (Spagna and Peattie, 2012; Wilson, 1967) (Fig. 1A,B; Movie 1). Therefore, we induced these spiders to drop two legs from a single tetrapod, a perturbation expected to cause maximal disruption of the expected default alternating tetrapod gait. In fact, tarantulas, wolf spiders and scorpions have been reported to adopt modified gaits in response to this autotomy treatment. These modified gaits include: (1) an ‘ablated tetrapod’ gait, in which they alternate using the intact tetrapod and a bipod formed by two legs remaining from the ablated tetrapod (Fig. 1C; Movie 1); or (2) a ‘modified tripod’ gait, in which they move the remaining six limbs in an adaptive version of the alternating tripod gait (Fig. 1D; Movie 1) (Bowerman, 1975; Wilshin et al., 2018; Wilson, 1967). These modified gaits have different trade-offs: adjusting to the modified tripod gait requires altering the phasing of leg motions, whereas using the ablated tetrapod gait requires moving with only two legs on the ground for part of each stride, causing potential instability. These prior findings provided us with models for studying how gait patterns change during recovery after autotomy.

We tracked the spiders' body and leg motions and used the results to compute measures of their locomotor performance. We then analyzed their leg motions during running using a novel combination of unsupervised learning and the gait space classification methods outlined in Wilshin et al. (2018) to compute the similarity between the measured data and proposed model gaits. Inspired by earlier work that used unsupervised learning to create ethograms from kinematic features (Hsu and Yttri, 2021), we applied clustering to the measured gait space data to identify both patterns and variability of the distribution of data in gait distance space. The goal of this alternative gait classification approach was to identify locomotory behaviors pre- and post-autotomy and regeneration that could either correspond to proposed gaits or new behaviors not previously reported (e.g. new gaits or novel compensatory mechanisms).

To determine how limb autotomy and regeneration influenced locomotor performance and gaits in spiders, we investigated the following hypotheses.

Hypothesis 1: spiders with missing and regenerated legs were expected to have impaired locomotor performance compared with intact specimens. We hypothesized that this would result in reduced running speed, greater path tortuosity (a measure of the deviation from a straight path), and more variable stride frequency and stride length.

Hypothesis 2: we proposed that tarantulas would be less statically stable when running with fewer legs or using regrown legs. We therefore hypothesized that they would widen their stance and increase their tarsal range of motion to enhance locomotor stability.

Hypothesis 3: we expected that spiders would respond to the destabilization imposed by leg loss by adopting modified gaits. We hypothesized that, consistent with previous studies (Wilshin et al., 2018; Wilson, 1967), spiders missing a diagonally opposed foreleg and hindleg would run with a combination of the ablated tetrapod and modified tripod gaits.

Hypothesis 4: spiders recovering from limb loss are expected to use either adaptive control or robust control if their neural control systems can maintain locomotor performance in the presence of these perturbations. We therefore hypothesized that, as suggested by prior studies, measures of running performance (e.g. speed, static stability) and observed locomotor gaits would exhibit a rapid recovery of stable locomotion by near-immediate adoption of a stable gait for robust control. Alternatively, recovery would correspond to a gradual, time-dependent restoration of locomotor performance, consistent with adaptive control.

## MATERIALS AND METHODS

### Animals

**Fig. 2.**
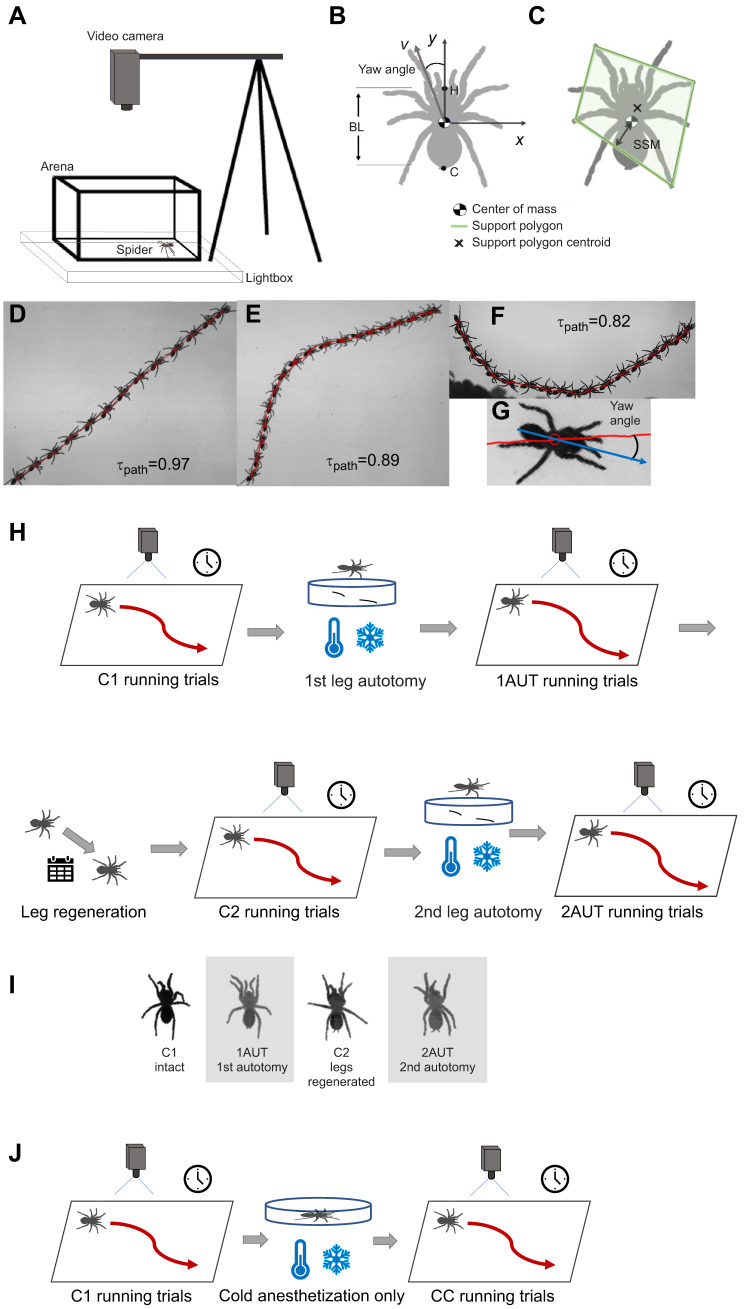
**Illustration of the geometry and treatments used for experiments, as well as the terminology used in tracking.** (A) Schematic diagram of arena and filming geometry during running trials. (B) Schematic illustration of the spider's body length (BL), center of mass (COM), yaw angle between the cranial–caudal axis and center of mass velocity (*v*), and *xy* axes used for tracking motion in the comoving body frame. (C) Illustration of the definition of the support polygon (green polygon) and static stability margin (SSM). (D–F) Superimposed images of running spiders filmed in dorsal view with the tracked body center of mass trajectory shown as a red line for different values of tortuosity (τ). (G) Sample image of the yaw angle deviating from the velocity direction. (H) Flow diagram showing steps in the protocol for the filming and experimental treatments. (I) Still images from video of the same specimen after each treatment in H; gray shaded regions indicated autotomized specimens. (J) Flow diagram of the cooling-only controls CC.

Captive-bred *Davus pentaloris* (Simon 1888) spiderlings with no prior history of limb loss (second to third instar, 3–5 months old) were purchased from a private breeder (Mikolaz Sarnecki, Prospiders, Warsaw, Poland) and imported in compliance with US Fish and Wildlife Service regulations. This species is not listed as endangered by CITES (Convention on International Trade in Endangered Species of Wild Fauna and Flora, 2025) or the IUCN Red List of Threatened Species (International Union for Conservation of Nature, 2025). We followed best practices for tarantula husbandry (Pellett et al., 2015) by housing specimens individually in an animal care room held at 25.6–27.8°C, 30% humidity, 12 h:12 h light:dark schedule, and feeding them cockroaches (*Blaptica dubia* and *Eublaberus posticus*). The body length (BL), defined as the distance between the anterior end of the chelicerae to the caudal end of the abdomen (Fig. 2B), was measured on a calibrated photograph for each day on which experiments were performed (median BL=16.0 mm, range=7.4–22.8 mm).

### Data analysis and statistics

All data analysis and statistics were performed using MATLAB v2023b (MathWorks, Natick, MA, USA) using the machine vision, curve fitting, statistics and machine learning toolboxes, unless stated otherwise; MATLAB functions are indicated below in italics. Differences between multiple means were computed using Kruskal–Wallis tests with *post hoc* correction using Dunnett's test (*kruskalwallis* and *multcompare* in MATLAB). Wilcoxon rank sum testing (*ranksum*) was used to compare paired measures. Linear regression was performed using *lmfit*. The reported *P*-values for linear regression fits were corrected using the Bonferroni–Holm *post hoc* test for multiple comparisons (Holm, 1979) using the MATLAB implementation ‘Bonferroni-Holm Correction for Multiple Comparisons’ (https://www.mathworks.com/matlabcentral/fileexchange/28303-bonferroni-holm-correction-for-multiple-comparisons). All testing used a corrected significance level of 0.05. Results are reported as means [95% CI]; s.d. was used as a measure of variability, unless the Kolmogorov–Smirnov test for normality was inconsistent with a normal distribution, in which case we report the median and median absolute deviation (MAD). Bootstrap calculations were performed using the MATLAB function *bootci* using 1000 bootstrap samples. Gait calculations involving circular statistics were performed using the MATLAB toolbox CircSTAT (Berens, 2009).

### Experimental procedure

Videos were filmed using a single camera (Photron FASTCAM model SA-3, San Diego, CA, USA; settings: 8-bit monochrome, resolution 216×928 pixels, 500 frame s^−1^, shutter 1/1000 s, working distance 80 cm). The spiders were filmed in dorsal view during running in an indoor arena consisting of a backlit clear, smooth acrylic box (19×27 cm) at ambient temperature (24.0±0.5°C) between 09:00 and 18:00 h (Fig. 2A). The video field of view showed spiders moving horizontally for a mean of six strides per trial (range=2–14 strides), where the stride period was defined as the time required for one full cycle of foot motion during locomotion, and a trial was defined as a single bout of locomotion from the start of running until the spider either stopped moving or reached the edge of the arena. Spiders were stimulated to move by approaching with a stick to provoke an escape response. When the camera's memory buffer reached capacity, the spider was covered with a small Petri dish to prevent movement while the video was saved in between filming bouts. The substrate was cleaned between each specimen to remove possible scent cues owing to silk and scent deposits (Persons et al., 2001).

Fig. 2H illustrates the various experimental treatments (Dataset 1, https://doi.org/10.6084/m9.figshare.28229174.v3). Control trials were performed on specimens with all legs intact prior to undergoing autotomy (C1). Spiders were then induced to autotomize in response to two legs (hindleg L4 and foreleg R1) being restrained as follows. First, the spider was anesthetized by placing it on a piece of cardstock on ice in a covered Petri dish. The dish was removed from ice once the spider was unresponsive to stimulation by touching (12–25 min), and the tarsus of each leg to be autotomized was adhered to the card using cyanoacrylate glue and then placed in the arena for the spider to recover. Upon recovery, the spider quickly shed the two restrained legs. All sequences of running starting immediately after autotomy were filmed until the spider stopped running (1AUT0). Next, the specimen was allowed to recover in its habitat for 19–24 h, then a second set of trials (1AUT1) was performed on the following day using the same filming protocol as the first day (1AUT1).

Afterward, the specimens were allowed to regrow the autotomized legs. Additional experiments were performed once the regenerated legs were fully developed (i.e. the same length, thickness and morphology as the intact leg on the opposite side). First, controls were filmed for spiders running using regenerated legs (C2, *N*=4), then specimens were induced to autotomize using the same protocol as the first autotomy. Finally, running trials were recorded on the day of the second autotomy (2AUT0, *N*=5) and 20–32 h after (2AUT1, *N*=5) (Table S2).

To test the effect of cold-anesthetization alone on spider locomotion, we first filmed a set of running control trials (C1, *N*=6) at ambient temperature on specimens that had not undergone autotomy. These specimens were then filmed running after being subjected to the same cooling treatment used before autotomy, but without inducing leg loss (cooled controls, CC; *N*=6) (Fig. 2J; additional details in Dataset 1, https://doi.org/10.6084/m9.figshare.28229174.v3).

### Video analysis and kinematics calculations

The anatomical features tracked on video included the spider body center of mass (COM), located between the abdomen and cephalothorax (Biancardi et al., 2011; Silva-Pereyra et al., 2019), the cranial and caudal ends, and the distal tips of all tarsi (Figs 1B, 2B; Table S4). The *xy* coordinates of each landmark were tracked by custom automated MATLAB code (Supplementary Material and Methods), and then semiautomated manual tracking in Direct Linear Transformation data viewer (DLTdv) (Hedrick, 2008) was used to correct mistracked and missing data (tracking uncertainty: ±0.8 mm in *xy* coordinates, ±2 deg for the cranial–caudal axis orientation). The spatial calibration (3.67±0.02 pixels mm^−1^) was measured from still images of a ruler; the error bar in the resolution included variation between the calibration measured from an image recorded on every day of experimentation as well as the uncertainty in the ruler length (15.00±0.05 cm) and pixel measurements (550±1 pixels).

For the majority of the C1 controls, CC cooling controls and autotomy treatments, a total of five trials per specimen were analyzed. The following exceptions were due to specimens becoming reluctant to run: (1) only four trials were recorded for one specimen's CC treatment; and (2) trials were recorded for only four of the five specimens for the C2 control after leg regeneration (five trials for two specimens, four trials for the other two).

The tracked coordinates were used to compute several kinematic measures to compare running performance before and after leg loss, leg regeneration and cooling. The velocity of the COM in the *xy* plane (**v**) and of each tarsus (**v**_i_) was computed from the tracked coordinates using a running quadratic fit (Movingslope, https://www.mathworks.com/matlabcentral/fileexchange/16997-movingslope) to the data over a time window of 50 ms for the COM and 40 ms for the tarsal coordinates; these times were chosen after initial testing found that smoothing the original data over these times using a running quadratic fit (*smooth* with the ‘rloess’ option) results in changes no greater than the tracking uncertainty. Previous research has found that spider running speed varies with body mass (*M*) as *v*_COM_∝*M*^0.353±0.08^ (Boehm et al., 2021) and that body mass scales with body length as *M*∝BL^2.70±0.04^ (Penell et al., 2018). Therefore, we expected that running speed should scale approximately linearly with body length: *v*_COM_∝BL^0.95±0.22^. In agreement with this, linear regression found that the measured mean running speed varied linearly with body length during control trials (*R*^2^=0.80; *v*=21.0 m s^−1^ [19.0, 23.0]×BL; slopes agreed at the 95% CI for C1 and C2 data). Consequently, relative speed (BL s^−1^) was reported to compensate for variability in body length, as in previous studies of spider locomotion (e.g. Spagna and Peattie, 2012; Silva-Pereyra et al., 2019). To look for evidence of steering failure, we measured path tortuosity using the straightness index τ_path_=*d*/*D*≤1, where *d* is displacement (i.e. distance between the initial and final COM positions) and *D* is total distance traveled along the trajectory (McLean and Skowron Volponi, 2018). In addition, to look for heading error, in which the body axis is misaligned with the direction of motion (Meshkani et al., 2023), we measured the yaw angle, defined as the angle between the instantaneous direction of COM velocity and the cranial–caudal axis (Wilshin et al., 2018) (Fig. 2B,G).

The motion of each intact tarsus was tracked to quantify gait kinematics. For each leg involved in locomotion, the gait cycle was defined as the time from when a specific tarsus first makes contact with the ground in a gait cycle until the instant before the same tarsus touches down in the next gait cycle. The stride period (*T*_stride_) and frequency (*f*_stride_=1/*T*_stride_) for each tarsus's motion were defined as the mean spacing of successive peaks in the tarsus's velocity along the COM motion. To show tarsal motion during locomotion, footfall diagrams were created for each time interval during which the COM speed was approximately constant (Fig. 5). Each tarsus's motion during a stride was divided into a ‘stance’ state, when the tarsus was fixed relative to the ground, and a swing state, when the tarsus moved relative to the ground. The stance state for each tarsus was estimated from the fraction of each gait cycle in which it moved caudally with respect to the COM; the swing state was then defined as the remainder of the gait cycle. The duty factor was estimated as the percent of frames in each gait cycle in which the tarsus in question was in stance. The stride frequency and stride length were computed for each tarsus, then averaged over all tarsi.

### Stability measures

We also computed several measures of static stability previously used in analyzing quasi-static locomotion (Ting et al., 1994). To do so, we first computed for each video frame the estimated number of tarsi in the stance state (*N*_support_) and the support polygon, defined as the convex hull of the *xy* coordinates of all tarsi in stance (Fig. 2C). For static stability, the support polygon must at minimum be a tripod (*N*_support_≥3) (Ting et al., 1994). The static stability margin (SSM) was determined from the minimum distance from the support polygon's edges to the projection of the COM onto the ground. For an organism at rest with respect to the ground that exerts only downward ground reaction forces, the system is statically stable when SSM>0 and in metastable equilibrium when SSM=0. If the centroid of the support polygon is at the same location as the COM, then SSM is at its maximum value, the ideal static stability margin (ISSM), and the system is said to be ideally statically stable; more generally, SSM≤ISSM. In the analysis of this study's data, we interpreted these stability measures cautiously because running tarantulas do not necessarily move quasi-statically. Even if SSM<0, they can still locomote stably by relying on dynamic stability mechanisms (Koditschek et al., 2004) or by grasping and adhering to surfaces (Silva et al., 2021).

To optimize stability, autotomized spiders could adopt an altered range of leg motions adjusted to cover the functional space of both the intact and missing tarsi. To explore this possibility, we measured the average angle between adjacent legs. If the legs responded to leg loss to remain uniformly spaced in angle around the COM, then the angle between adjacent legs should increase from 45 deg=360 deg/8 for intact specimens to 60 deg=360 deg/6 after autotomy, consistent with the measured values. The distribution of each tarsus's range of lateral and fore–aft motions provided a measure of whether neighboring tarsi filled in the functional space left vacant by an autotomized tarsus. To determine whether any tarsi [e.g. those adjacent to each missing leg (L3 next to autotomized L4 and R2 next to autotomized R1)] increased their range of motion to cover the functional space covered by two legs in the intact specimen, we plotted the position of each tarsus in a body-fixed frame with the +*y*-axis oriented along the COM velocity (Fig. 2B). In this frame, we measured the area swept out by the range of motion of each tarsus, after omitting outliers (i.e. values >3 MAD from the median), for intact and autotomized specimens. Third, because a wider stance provides a greater stabilizing torque for the same ground reaction force, we computed the leg extension, defined as the distance between the tarsus and the COM for each intact limb in the fore–aft and lateral directions pre- and post-autotomy.

### Gait analysis

Gait patterns were computed and then analyzed using unsupervised machine learning and compared with proposed model gaits: the alternating tetrapod (ALT) for intact spiders with eight legs, and the ablated tetrapod (ABT) and modified tripod (MT) gaits for autotomized spiders with six legs.

**Fig. 3.**
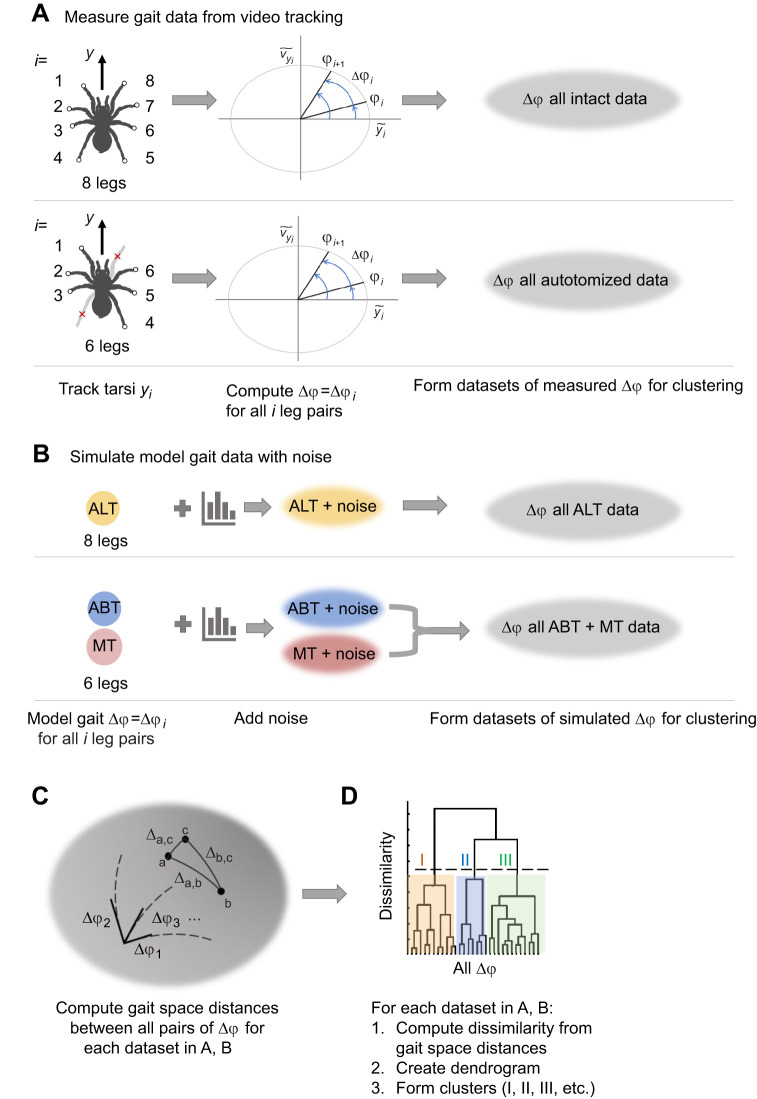
**Flow diagram illustrating the pipeline for calculating and clustering measured and simulated gait phase differences for autotomized treatments.** (A) Illustration of the calculation of the *i*th leg's phase (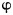
*_i_*; Eqn 1), the phase difference (Δ
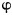
*_i_*) between the *i*th pair of adjacent legs (Eqn 2) from standardized values of the position (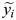) and velocity (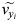) of each tarsus, and the formation of datasets for intact and separately for autotomy treatment data. (B) Simulated gaits were created with added noise for these models: alternating tetrapod (ALT), ablated tetrapod (ABT) and modified tripod (MT). The ABT and MT data then were combined to form a simulated autotomy dataset for clustering. Four datasets of phase differences were created for clustering as described in A and B. (C) These datasets of phase differences were then each used to compute the distances (Δ_AB_) between each pair of data points in the (*N*_legs_–1)-dimensional non-Euclidean gait space. (D) Finally, the distances were used to compute a dendrogram based on the dissimilarity between pairs of points. Clusters were assigned based on branches in the dendrogram with the greatest dissimilarities.

First, gait patterns were quantified using the methods developed in Revzen and Guckenheimer (2008) to compute the phase of oscillation of each leg during locomotion. This approach is a generalization of relationships for a simple harmonic oscillator moving periodically along the *y*-axis with constant amplitude *A* and frequency (ω). For the solution in which position, *y*, depends on time, *t*, as *y*=*A*cosω*t*, then the velocity and phase are given by 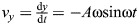 and 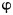
_*i*_(*t*)=ω*t*=atan(−*v*_*y*_/(ω*y*)), respectively. More generally, during locomotion, the position (*y_i_*) and velocity (*v_y_i__*) of the *i*th tarsus are oscillatory signals with noisy amplitude and frequency, and the effective oscillation phase can be estimated as:
(1)

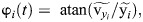
where position (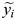) and velocity (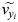) have been standardized to zero mean and unit variance (Revzen et al., 2013). (Fig. 3A) The difference in oscillation phase between each pair of intact adjacent legs (Δ
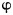
*_i_*) is computed as:
(2)

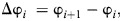
in which the intact legs are numbered *i=*1, 2, …, *N*_legs_ starting from the foremost left leg and running counterclockwise in dorsal view to the foremost right leg, as shown in Fig. 3A. Because 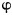, we define each gait using *N*_legs_–1 independent values of phase differences: 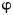
_gait_=(Δ
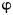
_1_, Δ
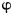
_2_,…, Δ
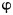
*_N_*__legs_–1_). For example, for an intact spider, the alternating tetrapod gait is defined by Δ
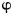
_ALT_=(Δ
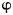
_1_, Δ
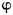
_2_,…, Δ
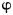
_7_), with all Δ
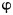
*_i_*=0.5 in a 7-dimensional gait space. Similarly, for autotomized spiders with six legs, the modified tripod has Δ
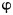
_MT_=(Δ
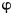
_1_, Δ
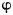
_2_,…, Δ
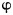
_5_) with all Δ
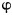
*_i_*=0.5 in a 5-dimensional gait space. The ablated tetrapod gait differs from the modified tripod in that the third phase difference is 0 instead of 0.5: Δ
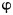
_ABT_=(Δ
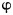
_1_, Δ
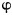
_2_,…, Δ
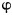
_5_)=(0.5, 0.5, 0, 0.5, 0.5).

To quantify measured gait patterns, the tracked values of the *i*th tarsus's position and velocity along the fore–aft direction (*y_i_* and *v_y_i__*) were used in Eqns 1 and 2 to compute the *N*_legs_−1 phase differences (Δ
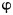
*_i_*) for each frame in units of oscillation cycles. Next, these data were analyzed using their positions in a metric gait space with gait coordinates Δ
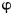
_gait_=(Δ
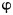
_1_, Δ
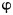
_2_,…, Δ
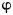
*_N_*__legs_–1_) (for full details, see Wilshin et al., 2017, 2018). Because each of the Δ
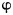
*_i_* is periodic with range [0, 1] cycle, each gait corresponds to a point on an (*N*_legs_–1)-dimensional hypertorus. (Fig. 3D) The distance between two points in this gait space (*d*_AB_), corresponding to two different gaits, A (Δ
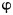
_A_) and B (Δ
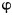
_B_), depends on the minimum distance (δ_Ab*i*_) along each dimension Δ
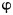
*_i_* for all *i*=1, 2, …, (*N*_legs_–1) leg pairs:
(3)

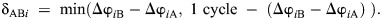
This is because each value of δ_AB*i*_ can be thought of as the minimum distance between two points on a circle with unit circumference. As explained in Wilshin et al. (2018), the total distance *d*_AB_ between points A and B in gait space then can be computed from the set of δ_AB*i*_ for all *i* leg pairs using a metric tensor, *g_ij_* (Table S1), that accounts for the mean phase of all legs during a gait cycle and ensures each *i*th leg pair's phase difference is equally weighted. The gait distance (*d*_AB_) in cycles is then defined by:
(4)

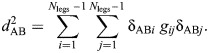
The maximum distance possible between two points in gait space, *d*_max_=max*d*_AB_, occurs when δ_AB*i*_=0.5 cycle for all *i* (Table S1). We used this fact to compute a normalized gait distance Δ_AB_ that has range [0,1]:
(5)

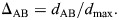
For example, the modified tripod and ablated tetrapod gaits are separated by a distance Δ_ABT–MT_=0.29 of the maximum possible distance. To compare a dataset of measured phase differences (e.g. all Δδ*_i_*_frame_ for each frame of each trial for a given treatment) with the Δ
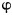
*_i_*_gait_ for a model gait, we used Eqn 5 along with bootstrapping to measure the mean and 95% CI of the gait space distances between all measured and model phase differences. This allowed us to compare, for example, gait patterns measured for intact spiders with the ideal alternating tetrapod gait, and those for autotomized spiders with both the ideal ablated tetrapod and modified tripod gaits.

For gait classification, we next used unsupervised learning with the gait space distances as follows. First, a merged dataset was formed from all measured leg phase differences (Δ
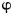
*_i_*) for all the trials under consideration. Next, the gait space distances between each pair of Δ
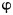
*_i_* values were computed using Eqn 5. A dendrogram was created from these results using hierarchical agglomerative clustering and Ward's linkage (*linkage*, *dendrogram* and *cluster* in MATLAB) with a similarity measured based on the pairwise gait space distances (Fig. 3C,D). The dendrograms created in this way display the height (dissimilarity) derived from the gait distance computed for each pair of leaves shown at the bottommost end of each branch; here, a leaf is a measured set of Δ
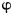
*_i_*, for all *i* leg pairs (Fig. 3D). Each branch corresponds to a cluster of gait patterns with varying levels of similarity. To find the clusters with the greatest dissimilarity, we start at the top of each dendrogram and find the branch points at the greatest height. This allowed us to group together more or less similar values of Δ
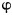
*_i_* without reference to predefined gaits. This procedure was performed separately for datasets of intact and autotomy treatments to determine how their data clustered in gait space independently. For each of these datasets, we also used bootstrapping to compute the mean and 95% CI of the distance in gait space between each cluster in the measured data and the model gaits expected for that dataset.

To validate using unsupervised learning to group noisy measured data, we also performed clustering on synthetic gait data with simulated noise prepared as follows. We started by creating a perturbed gait dataset for each of the ideal model gaits (Fig. 3B). For each model gait, we generated 1000 random samples of Δ
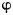
*_i_* with added noise using the von Mises distribution (*circ_vmrnd*; Berens, 2009) with the parameter μ*_i_* that characterizes the average equal to the Δ
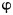
*_i_* values for the model gait and the mean dispersion parameter κ determined from fitting the measured phase difference data for the intact specimens and separately for the autotomized specimens to a von Mises distribution. The simulated perturbed ALT data then were compared with the results for the intact measured data. To create a simulated dataset for comparison with the measured autotomy data, we merged the perturbed ablated tetrapod and modified tripod samples into a single dataset (Fig. 3B). These two simulated sets of perturbed data were then analyzed using the gait space distance and clustering methods described above (Fig. 3C,D).

The results of clustering the measured and simulated data were used to estimate the effect of noise on both phase differences and gait space distances. This approach allowed us to explore whether the leg motions during locomotion corresponded to a series of successive motions corresponding to a hypothesized model gait, a different, but still regular, gait pattern, or a noisy series of leg motions not corresponding to a fixed gait pattern per se. We also used the clustering results to compare statistics for the speed, the SSM and the number of feet in stance for the different gait patterns identified.

To illustrate how the gait clustering results corresponded to dynamic leg motions, we created videos that showed the spider in a frame of reference with the COM fixed and the vertical aligned with the fore–aft direction, with the tarsi in stance in each frame indicated by a support polygon (Movie 1). For each frame, we also plotted a label indicating the cluster assignment at that time, and colored the support polygon using a code based on the most similar set of comoving tarsi for the corresponding model gait (Fig. 1B,C).

## RESULTS

This study investigated individual recovery in response to autotomy, rather than population level effects. Consequently, we collected a high-resolution dataset with large amounts of data per specimen and treatment. All kinematic and gait analyses were performed on a total of 43,562 frames, equivalent to 96–173 strides per treatment for seven treatments and *N*=5 specimens (*N*=7 for controls for the effect of cold anesthetization) (Dataset 1 in https://doi.org/10.6084/m9.figshare.28229174.v3). This number of specimens and strides per treatment is comparable to or greater than that used in prior detailed studies of spider locomotion (e.g. Hao et al., 2020; Shamble et al., 2017; Silva-Pereyra et al., 2019; Wilshin et al., 2018).

### Running performance

The spiders consistently ran without stumbling or falling, as shown in the typical videos for C1, C2 and 1AUT0 treatments in Movie 1. We first consider whether various locomotion measures depended on the time after treatment. Body yaw angle, tortuosity, stride frequency, duty factor and SSMs did not depend significantly on time in any trials, and running speed had a significant correlation with time in only 1 of 24 trials (statistics in Dataset 1, https://doi.org/10.6084/m9.figshare.28229174.v3). Therefore, we pooled together the data for each trial across times before performing further analysis.

**Fig. 4.**
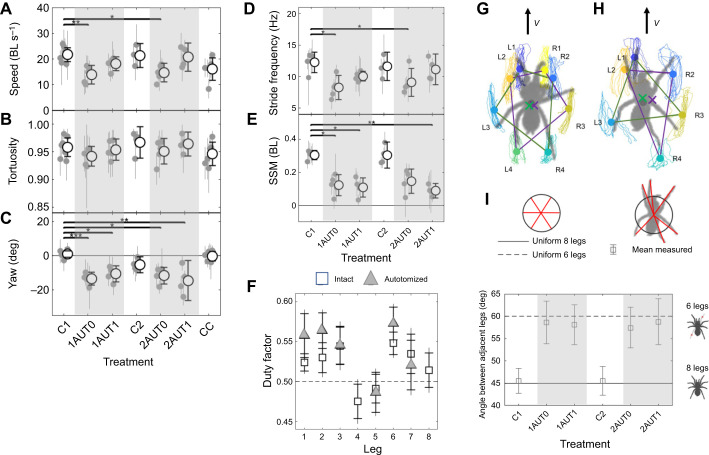
**Kinematic and posture measurements among treatments.** (A–E) Summary statistics for each variable for each treatment as grand means or medians and 95% CI (black open circles and error bars); gray filled markers and error bars show means or medians and 95% CI for each specimen (for each treatment, *N*=5 specimens, *n*=5 trials per specimen). Gray shaded regions correspond to autotomy treatments. Treatments that are significantly different from the controls C1 as indicated by Kruskal–Wallis testing with correction are indicated by lines and asterisks (*P≤0.05, **P≤0.01, ***P≤0.001). (F) The duty factor for each leg for the intact and autotomy treatments [grand means, error bars: 95% CI (with caps) and s.d. (no caps)]. (G,H) Examples of typical tarsal range of motion during locomotion. Colored lines and markers indicate measured tarsus range-of-motion trajectories and motion centroids for (G) an intact specimen with eight legs (C1 control) and (H) the same specimen with six legs the day after the first autotomy treatment (1AUT1). The distal end of each tarsus is plotted in a body-fixed reference frame in which the body COM (gray circle) is at the origin and the velocity (**v**) is oriented vertically. The mean positions of each tarsus (colored circles) in the corresponding tetrapods (G) and tripods (H) are shown as purple and green×markers. (I) Comparison of the angle between adjacent legs for each treatment expected if legs are uniformly distributed in angle about the COM (solid circles) with the measured grand mean and 95%CI (open circles and error bars).

We next tested the effect on running performance of the two successive two-leg autotomies and subsequent regeneration. Fig. 4A–C shows the results of comparing the mean speed, tortuosity and yaw for the control C1 with other treatments (for full statistics, see Tables S2, S3). Tarantula running speed did not differ significantly between controls conducted before and after leg regeneration (C1 versus C2). There also was no reduction in speed associated with autotomy: although speed and stride frequency were reduced relative to the control on the day of autotomy (but not the day after), this effect was not significantly different from the decrease owing to cold anesthetization alone (CC) (Fig. 4A,D). There were no significant differences in stride length among treatments (Fig. S1D).

We separately tested whether the spiders had improved running performance for treatment 2AUT0 compared with 1AUT0, consistent with their learning from the first autotomy, using Wilcoxon ranked sum testing between paired treatments of speed, tortuosity and yaw grouped by specimen. None of these measures differed between the first and second autotomy.

The path tortuosity, τ_path_, did not differ significantly among any treatments. (Fig. 4B) The tortuosity varied over a narrow range close to the maximum value of 1 for a straight line (135 values in the range [0.896, 0.993], one outlier at 0.82) (Fig. 2D–F).

Fig. 4E shows that the SSM decreased significantly in all but one autotomy treatment. For intact and autotomized treatments, the duty factor exceeded 0.5 (i.e. the tarsus was in contact with the ground for over half the stride cycle on average) for all but the hindmost legs (i.e. legs *i*=4 and 5; see Fig. 4F). This is because the hindlegs frequently drag (see videos for the intact and regrown cases in Movie 1); during dragging, tarsi that rest on the ground move at the same speed as the COM, thereby lowering the computed duty factor (which depends on foot motion relative to the COM), even though the legs being dragged may be able to support weight.

We next consider evidence for postural changes post-autotomy. After autotomy, the specimens ran with their bodies rotated with yaw of 11–15 deg relative to the forward velocity, in contrast to the zero yaw angle observed for pre-autotomy controls (Fig. 4C). See Fig. 4G,H for a typical example of how the motions and mean orientations of the different legs compared before and after autotomy. After autotomy, the spiders also increased the mean angle between each pair of adjacent legs to maintain a uniform leg spacing for both intact and autotomized specimens (Fig. 4I). Finally, Fig. S1A–C shows that neither autotomy nor leg regeneration resulted in significant differences in the tarsal range of motion or lateral and fore–aft leg extension, with the exception of a 1.3× increase in the range of motion after the first autotomy (Fig. S1B,C).

### Gait analysis

**Fig. 5.**
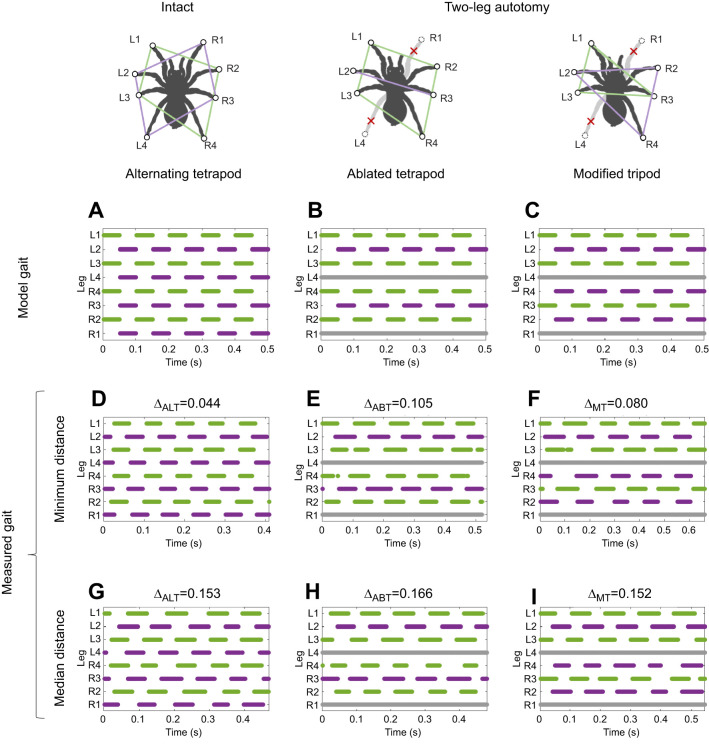
**Gait footfall diagrams for idealized models and measured data.** The corresponding pods (sets of coordinated legs) are shown schematically at the top as polygons in colors matching their hypothesized motion; gray lines indicate missing data for autotomized legs. Simulated model gaits are shown in A–C. Examples computed from empirical data are shown for five strides per trial for trials with minimum gait distances (D–F) and for trials with the grand median distance from the closest model gait (G–I). The gait space distance to the closest model gait is given above each diagram. The corresponding treatments were: C1 for D,G; 1AUT0 for E,F; 1AUT1 for I; and 2AUT1 for H.

Fig. 5 shows footfall diagrams for the idealized gaits proposed for the intact and autotomized spiders and for measured data for trials with the minimum and median gait distances to each idealized gait. To explore these gait patterns further, we consider the results of clustering data from the gait space analysis for the simulated and measured phase differences. First, we found that the synthetic data created by adding noise to the ablated tetrapod and modified tripod were correctly grouped with the original model gait by clustering for 95% of samples; assignment by closest gait distance was correct in 92% of samples. Given this validation of the accuracy of clustering applied to simulated gait data, we next applied this method to measured data.

**Fig. 6.**
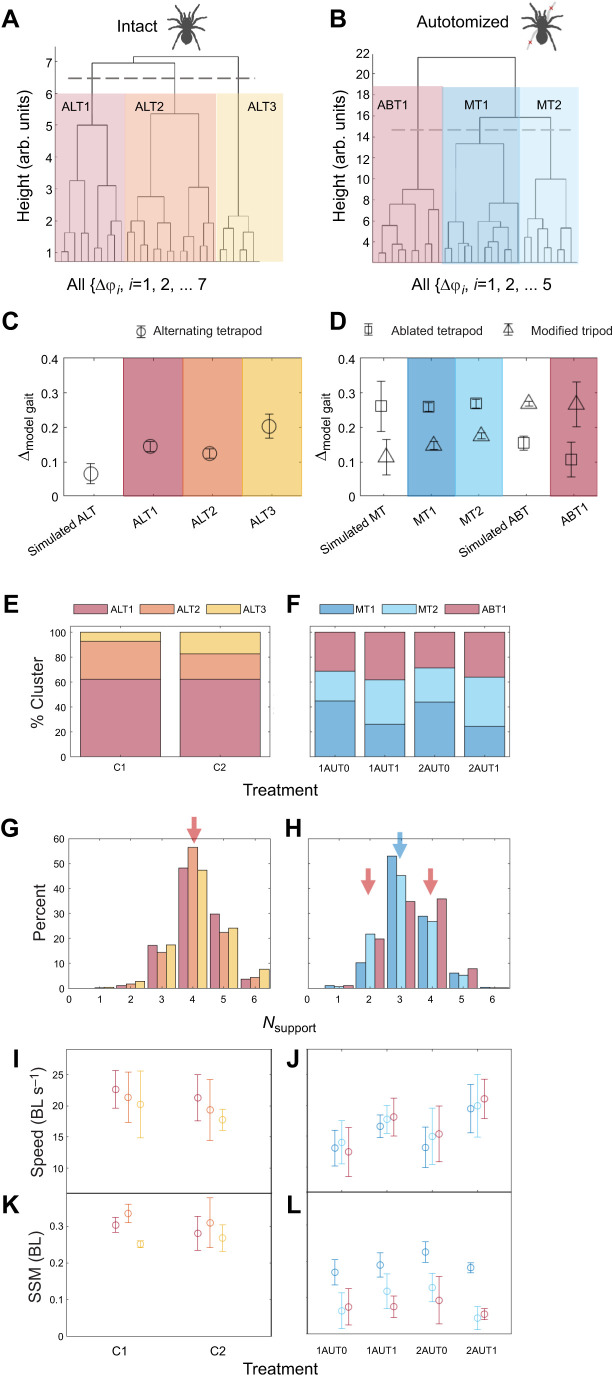
**Gait space analysis.** Dendrograms for (A) all intact (C1 and C2 control) data and (B) all autotomized treatment data (1AUT0, 1AUT1, 2AUT0, 2AUT1). Leaves in the dendrogram correspond to measured phase space differences, while heights correspond to dissimilarity between the data computed using gait space distances. Clusters are labeled by the most similar model gait: ALT1, 2, 3 for alternating tetrapod, ABT1 for ablated tetrapod, and MT1, 2 for modified tripod. Comparison of gait space distances from the ideal model gaits for (C) intact and (D) autotomy simulations and measured gait clusters (grand means, s.d. error bars) (for each treatment, *N*=5 specimens, *n*=5 trials per specimen). (E,F) Fractions of the data grouped into the clusters shown in A and B by treatment averaged over specimens. (G,H) Histograms indicate the percent of time for which each number of tarsi was in stance (*N*_support_) by cluster for all intact and all autotomized treatments. Colored arrows indicate the expected values for the relevant model gaits: magenta at *N*_support_=4 for the alternating tetrapod in G, and *N*_support_=2 and 4 for the ablated tetrapod in H, blue for the modified tripod in H. (I–L) Comparison of the (I,J) speed (grand mean, s.d. error bars) and (K,L) SSM (grand median, MAD error bars). Marker and bar colors in I–L agree with those used in C–H.

We created two datasets for the cluster analysis by pooling the gait space data for (1) all intact control trials (C1, C2) and (2) all of the autotomy trials (1AUT0, 1AUT1, 2AUT0, 2AUT1), because Kruskal–Wallis testing showed no significant differences among treatments (Dataset 1 in https://doi.org/10.6084/m9.figshare.28229174.v3). Fig. 6A and B show the two dendrograms created by applying hierarchical clustering to each of these empirical phase difference datasets. We defined the three most dissimilar clusters for the intact data based on the branch point between cluster ALT3 and a second branch that splits into clusters ALT1 and ALT2 (Fig. 6A). [The names for these and all other gait clusters were based on their proximity between the clusters' centroids and the relevant model gaits; e.g. for intact data, clusters ALT2, ALT1 and ALT3 have centroids that are 11.5%, 14.6% and 20.4% of the maximum possible distance from the alternating tetrapod (Fig. 6C).] Similarly, the dendrogram for the autotomy data first branches into cluster ABT1 and a second branch, which further splits into the branches labeled MT1 and MT2 (Fig. 6B; statistics in Dataset 1 in https://doi.org/10.6084/m9.figshare.28229174.v3). Based on the similarity of the gait space and kinematic analysis of clusters ALT1 and ALT2 and clusters MT1 and MT2 for the intact and autotomy data, respectively, described below, we did not further subdivide the data. For the autotomy data, the MT1 and MT2 clusters are closer to the simulated modified tripod (MT) data, and the ABT1 cluster is closer to the ablated tetrapod (ABT) simulation (Fig. 6D).

The distributions of clusters are shown in Fig. 6E,F; chi-squared testing indicated significant differences in the distributions among all treatments (Dataset 1 in https://doi.org/10.6084/m9.figshare.28229174.v3). The majority (62%) of the intact data corresponded to ALT1, with the distribution between the other clusters changing from 7.5% of C1 in ALT3 to 17.5% of C2 (Fig. 6E). The majority of gait data for autotomized treatments (Fig. 6F) was predominantly associated with clusters similar to the modified tripod (MT1 and MT2). To test whether learning resulted in the spiders adopting gaits similar to the modified tripod to a greater extent at later times, we compared the fraction of data clustered as either MT1 or MT2 (MT clusters) for autotomy treatments at different times. The fraction of data in MT clusters decreased by 7.3% from the first to second day of each autotomy treatment (i.e. from 1AUT0 to 1AUT1 and from 2AUT0 to 2AUT1), but slightly increased (2.2%) between the first and second autotomy treatments on either day (i.e. from 1AUT0 to 2AUT0 and from 1AUT1 to 2AUT1).

Fig. 6I,J shows that the speed for each cluster was similar among treatments. In particular, the speeds for each cluster in the autotomy data were similar for the 1AUT0 and 2AUT0 treatments, indicating no effect of learning owing to prior experience with autotomy. Comparing the SSM among clusters revealed that the MT2 and ABT1 clusters had a lower SSM than MT1 (Fig. 6K,L). Distributions for treatments for intact specimens have a dominant peak at *N*_support_=4, as expected for the alternating tetrapod (Fig. 6M). For autotomy treatments (Fig. 6N), the MT1 and MT2 cluster data had a single peak at *N*_support_=3, the value expected for the modified tripod gait. However, the ABT1 cluster had its most prominent values at *N*_support_=3 and 4; although a value of 4 was consistent with one intact tetrapod, the peak at 3 disagreed with the value of 2 expected for the bipod formed by the ablated set of two tarsi. In general, none of the data for any treatment agreed with an aerial phase (i.e. intervals during which *N*_support_=0 and hence no tarsi are in contact with the ground).

**Fig. 7.**
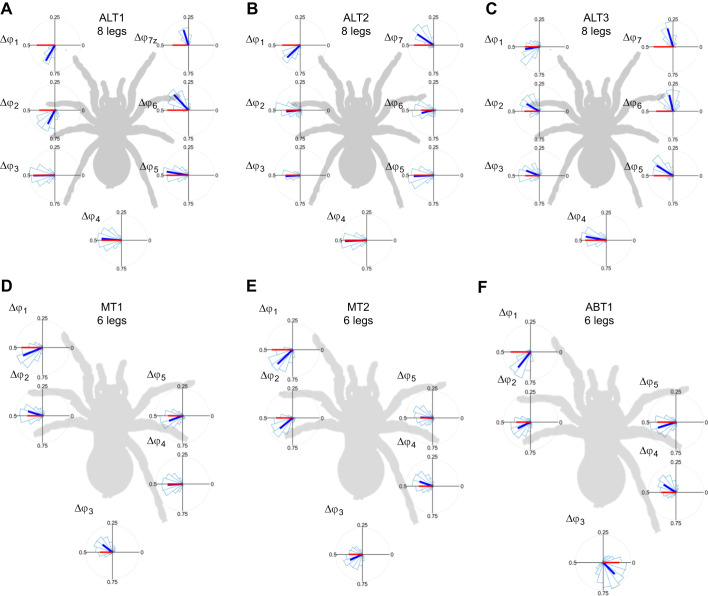
**Polar plots of measured leg pair phase difference histograms.** Circular statistics are shown for the leg pair phase differences, for (A–C) the three clusters (ALT1, ALT2, ALT3) for all the intact data and (D–F) the three clusters (MT1, MT2 and ABT1) for all the autotomy treatments. Polar plots of histograms (light blue open bars) of the distributions of the phase differences (Δ
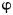
*_i_*) between pairs of adjacent legs for all trials for all autotomized trials grouped into the three clusters indicated in A and B. In each plot, the direction of the circular mean vector is shown by a blue solid line; a red solid line corresponds to the direction of the most similar model gait. The length of both lines indicates the amount of dispersion of the measured Δ
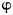
*_i_*, with shorter lines corresponding to greater dispersion. If the direction and length of the blue and red lines agree, this indicates good agreement between the measured and model gaits (statistics in Dataset 1 in https://doi.org/10.6084/m9.figshare.28229174.v3).

Fig. 7 shows the distribution of measured phase differences (Δ
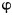
*_i_*) for each leg pair for each cluster. In the intact data, the hindmost legs (Δ
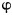
_3_, Δ
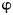
_4_, Δ
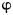
_5_) moved out of phase by 0.5 cycles, in agreement with the alternating tetrapod. However, for each ipsilateral pair of forelegs (i.e. Δ
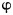
_1_, Δ
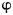
_2_, Δ
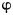
_6_, Δ
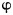
_7_), the phase of the more cranial of each pair (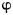
*_i_*) lagged that of the other leg.

For the autotomized data, the phase differences between remaining leg pairs (Δ
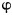
_1_, Δ
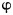
_2_, Δ
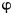
_4_) were similar to the values in the intact data. The phase differences affected by an autotomized leg (Δ
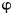
_3_, Δ
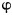
_5_) were in close agreement with values for the relevant model gaits. In general, there were similar spreads for all leg pairs for all treatments and clusters, with the exception of a larger spread in Δ
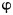
_3_ between the legs adjoining the autotomized hindleg for the ABT1 cluster.

The videos annotated with the gait cluster assignments in Movie 1 show that these variable gait patterns do not conform to two stable sets of legs alternately placed in stance. They also show that for the autotomy treatments, frames in which only a statically unstable bipod or monopod were in stance often were followed quickly by more cranially oriented tarsi being planted to form a support polygon.

## DISCUSSION

Juvenile tarantulas that had never experienced autotomy were found to achieve a stable gait immediately after 25% leg loss and to resume their pre-autotomy speed within 1 day. In addition, two-leg autotomy following cold anesthetization did not result in a speed significantly lower than that owing to cold anesthetization alone in the hour after treatment; this lack of an additional effect owing to leg loss was surprising because the autotomy treatment was designed to maximally impact their normal gait pattern. Furthermore, the spiders did not increase their path tortuosity in response to leg loss, unlike harvestmen and water-walking insects (O'Neil et al., 2024; Escalante and O'Brien, 2024). Although autotomy did not affect the extension or range of motion of their legs, the spiders did modify their posture so as to move with non-zero body yaw and more widely spread legs. Overall, this indicates that these spiders achieved a rapid recovery of running performance even faster than the 2 days found for autotomized harvestmen (Escalante et al., 2020) and comparable to that found for water-walking insects in O'Neil et al. (2024).

A comparison of data for the first and second autotomy treatments did not support a role for learning in this recovery: there was no significant difference between kinematic measures and only a small (2.2%) change in gait use (i.e. between gaits similar to the more stable modified tripod versus less stable ablated tetrapod). These results agree with a picture in which these spiders responded to the loss of legs by rapidly adjusting their locomotion to achieve a new stable gait (robust control), rather than using error-based learning (adaptive control) (Mongeau et al., 2024).

The costs and benefits of both autotomy and regeneration, and hence their ecological implications, depend on how well the regrown appendage functions for its intended purposes (Jagnandan et al., 2014; Maginnis, 2006). It is therefore of interest that spiders with regenerated legs ran at the same speed after as before autotomy, as found for purple shore crabs (Prestholdt et al., 2022), as well as exhibiting no significant difference in posture, path tortuosity, stride frequency or length, or SSM, using gaits similar to those used by intact specimens. This strikingly full recovery of locomotor performance indicates that these spiders were able to regenerate fully functional legs after autotomy, similar to the finding that some species of spiders are able to weave functional webs using one or two regenerated legs within 24 h of molting (Vollrath, 1990).

The gait clusters found using unsupervised learning were close in gait space to the ideal model gaits proposed for intact and autotomized spiders, indicating that they had similar mean phase differences between adjacent legs (Wilshin et al., 2018; Wilson, 1967). However, these clusters also exhibited additional structure in the measured gait patterns that differed from these models. For example, the legs did not move in two synchronized groups as predicted by the model gaits: all of the identified clusters had several adjacent leg pair differences that disagreed significantly with the 0.5 or 0 cycle offsets predicted for the model gaits. These variations resulted in more than one cluster resembling a model gait in some cases. For example, in the gait clusters found for intact spiders, the alternating tetrapod gait described the phase differences between the ipsilateral and contralateral pairs of adjacent hindmost legs, but not those between the foremost leg pairs. In another example, the autotomized data had two clusters that resembled variants of the modified tripod (MT1 and MT2) with different timing of leg motions, as well as different SSMs.

Prior research has shown that arthropod locomotor gaits have complex, dynamic phase difference distributions that are only approximately similar to the alternating tetrapod and other models (Biancardi et al., 2011; Silva-Pereyra et al., 2019; Spagna and Peattie, 2012; Weihmann, 2013, 2020; Wilson, 1967). This variability in gaits becomes particularly noticeable when locomotion is significantly perturbed, e.g. for movement on rough terrains by dogs (Wilshin et al., 2017) and by limb loss in arthropods (Han et al., 2024; Hughes, 1957; O'Neil et al., 2024).

Here, the use of clustering to group leg motions enabled us to identify patterns in the gait data corresponding to differences in both regular coordination and in variability in the timing of leg motions. This showed that the spiders studied here adapted to leg loss by adopting gaits not well described by a fixed pattern of leg motion. For example, measured phase difference distributions (Fig. 7) indicate that for most clusters, the measured interleg coordination patterns between pairs of the hindmost legs were the most consistent with values expected for the corresponding model gaits, and those involving the forelegs were the least consistent. These differences in leg coordination may relate to differences in leg use: forelegs play a role in pulling, sensing and deceleration, whereas hindlegs primarily propel and stabilize (Pullar and Paulin, 2018 preprint). We also observed the spiders dragging one or more hindlegs, which may serve to enhance stability (Spagna et al., 2011; Weihmann et al., 2015).

In addition, the distribution of feet in stance at any time did not follow expectations from model gaits in several ways. For all measured gait clusters, there was a broad distribution of the number of feet in stance, instead of well-defined peaks at the values expected for the most similar model gaits. This resulted in the foremost legs having measured duty factors >0.5 due to having more legs in stance at any given time than predicted for alternately moving synchronous sets of feet. The ablated tetrapod gait (ABT1) most frequently had three and four feet in stance, in disagreement with the ablated tetrapod model prediction of a 50:50 frequency of the statically unstable bipod and stable tetrapod.

These findings have implications for locomotion after autotomy. Interestingly, even though both the ABT1 and MT2 clusters had lower SSMs than MT1, all three measured post-autotomy gait clusters had similar speeds. The difference in SSM was due to the ABT1 and MT2 clusters having a higher instance of two or fewer tarsi on the ground. This lack of a dependence on static stability can be explained if these spiders are dynamically stable (Hof et al., 2005). For example, humans can walk stably with crutches using the swing-through gait, undergoing stable inverted pendulum motion about a pivot point formed alternately by the two crutch tips and the ‘good leg’ (Rasouli and Reed, 2020). Supporting this interpretation, the annotated videos for autotomized specimens show similar behavior in which short sequences of a statically unstable bipod are followed by a stable support polygon (Movie 1); in these cases, the bipod serves as a temporary pivot point about which the spider rotates quickly to plant additional feet on the ground to maintain stable motion, the behavior referred to as ‘limping’ in Wilshin et al. (2017). This behavior is frequent enough that spiders moving using the ABT1 cluster phasing have two or fewer tarsi on the ground only <27% of the time, significantly lower than the 50% predicted by the ideal ablated tetrapod gait, although greater than the <2% measured for intact specimens.

More generally, these results show that gait space clustering can identify patterns in empirical data without reference to predefined gaits, allowing for both the matching of measured gaits with known patterns and the discovery of new locomotor behaviors. This meets a pressing need in the study of locomotion by multi-legged animals, for which automated tracking now facilitates the collection of extremely large datasets of leg motions that are not readily interpretable using standard methods. To facilitate applying these methods in other contexts, we have provided simplified code at https://github.com/amadorkane/Gait-space-clusterer-for-multilegged-locomotion.git for analyzing the gait space distances and performing clustering on phase data for four-, six- and eight-legged locomotion that can be easily modified to work with varying degrees of leg loss. This approach could be used to determine how locomotion varies during different behaviors (e.g. moving at different speeds or orientations, carrying loads, pursuing prey or displaying) or in response to other challenges, such as differences in terrain or changes in environmental conditions.

Phase difference gait data alone cannot identify behaviors such as leg dragging, leg joint angle dynamics, internal and vertical body motions, and other movements that enable dynamic stability, so ideally it should be analyzed in combination with kinematic data and annotated video. In future work, these methods can be modified to work with three-dimensional data for leg and body motions facilitated by deep learning methods for automatically tracking motion on video (O'Neil et al., 2024). This should enable, for example, relating changes in gait to the mechanical work performed during locomotion (Silva-Pereyra et al., 2019), the effect of leg loss on energetics of locomotion (Escalante and Elias, 2021), and how animals redistribute loads among their remaining legs (Meshkani et al., 2023) in response to leg loss.

The comparative biomechanics of limb autotomy is also an important source of inspiration for legged robot design. Past studies of how robots can achieve stable gait coordination with one or more damaged or missing legs have used arthropod-inspired control algorithms and sensors that allow reprogramming gait phase relationships as well as body and leg posture (Görner and Hirzinger, 2010; Mostafa et al., 2010; Schilling et al., 2007; Shih et al., 2012; Spenneberg et al., 2004; Zhang et al., 2024), and leg compliance (Görner and Hirzinger, 2010). Using clustering to provide a deeper understanding of actual gait patterns during animal locomotion promises further insights relevant for robotics.

In conclusion, we found that intact and autotomized spiders both ran at similar speeds using variable gait patterns that only approximated model gaits in which legs move in rigidly synchronized sets. This study also found that that body orientation interacts with leg use, with an increased body yaw facilitating the redistribution of leg angles required to adapt the limb kinematics to compensate for leg loss. Together, these adaptations appear to render these spiders robust to the perturbations caused by leg loss, and thereby likely more responsive to similar challenges encountered during routine locomotion, such as uneven terrain, missed footing and using some legs for non-locomotory purposes (e.g. load-bearing or sensing). These results are relevant for understanding how arthropods cope with limb loss and for designing fault-tolerant locomotion in robotics.

## Supplementary Material

10.1242/jexbio.250243_sup1 Supplementary information
